# Circadian clock does not play an essential role in daylength measurement for growth-phase transition in *Marchantia polymorpha*

**DOI:** 10.3389/fpls.2023.1275503

**Published:** 2023-11-08

**Authors:** Yuki Kanesaka, Keisuke Inoue, Yuki Tomita, Shohei Yamaoka, Takashi Araki

**Affiliations:** ^1^ Graduate School of Biostudies, Kyoto University, Kyoto, Japan; ^2^ Center for Living Systems Information Science, Graduate School of Biostudies, Kyoto University, Kyoto, Japan

**Keywords:** circadian clock, daylength, growth-phase transition, *Marchantia polymorpha*, photoperiod, phytochrome

## Abstract

Daylength is perceived as a seasonal cue to induce growth-phase transition at a proper time of a year. The core of the mechanism of daylength measurement in angiosperms lies in the circadian clock-controlled expression of regulators of growth-phase transition. However, the roles of the circadian clock in daylength measurement in basal land plants remain largely unknown. In this study, we investigated the contribution of circadian clock to daylength measurement in a basal land plant, the liverwort *Marchantia polymorpha*. In *M. polymorpha*, transition from vegetative to reproductive phase under long-day conditions results in differentiation of sexual branches called gametangiophores which harbor gametangia. First, we showed that a widely used wild-type accession Takaragaike-1 is an obligate long-day plant with a critical daylength of about 10 hours and requires multiple long days. Then, we compared the timing of gametangiophore formation between wild type and circadian clock mutants in long-day and short-day conditions. Mutations in two clock genes, Mp*TIMING OF CAB EXPRESSION 1* and Mp*PSEUDO-RESPONSE REGULATOR*, had no significant effects on the timing of gametangiophore formation. In addition, when *M. polymorpha* plants were treated with a chemical which lengthens circadian period, there was no significant effect on the timing of gametangiophore formation, either. We next observed the timing of gametangiophore formation under various non-24-h light/dark cycles to examine the effect of phase alteration in circadian rhythms. The results suggest that daylength measurement in *M. polymorpha* is based on the relative amount of light and darkness within a cycle rather than the intrinsic rhythms generated by circadian clock. Our findings suggest that *M. polymorpha* has a daylength measurement system which is different from that of angiosperms centered on the circadian clock function.

## Introduction

To increase reproductive fitness, it is crucial for plants to undergo the growth-phase transition from vegetative to reproductive phase at the suitable season with favorable environmental conditions. Many plants measure daylength as the most reliable seasonal cue to ensure the proper timing of the growth-phase transition. According to the external coincidence model, daylength measurement is based on the interaction of the endogenous circadian clock with the environmental light signal (Bünning, 1936; Pittendrigh and Minis, 1964). The circadian clock sets a light-sensitive phase by regulating the expression of some key regulators. When light signal coincides with the light-sensitive phase, daylength-dependent response is induced. The mechanism of daylength measurement based on the external and internal coincidence models in plants is best studied in *Arabidopsis thaliana* (*Arabidopsis*) (Sung et al., 2015). *Arabidopsis* is a facultative long-day (LD) plant, in which flowering is promoted under LD conditions, but eventually occurs under short days as well. In *Arabidopsis*, the timing of the expression peak of *CONSTANS* (*CO*), the transcriptional activator of a florigen gene *FLOWERING LOCUS T* (*FT*), is of critical importance for daylength measurement (Suárez-López et al., 2001). The *CO* expression is regulated by *GIGANTEA* (*GI*) and *FLAVIN*-*BINDING KELCH DOMAIN F-BOX PROTEIN 1* (*FKF1*). Both *GI* and *FKF1* expression is regulated by the circadian clock, and the two proteins interact with each other in the presence of blue light. The GI-FKF1 complex degrades transcriptional repressors, CYCLING DOF FACTORs (CDFs), thereby allowing *CO* transcription (Imaizumi et al., 2003; Imaizumi et al., 2005; Sawa et al., 2007). Under LD conditions, the coincidence of high expression of *CO* and light induces the expression of *FT* (Roden et al., 2002; Yanovsky and Kay, 2002; Valverde et al., 2004).

Mutations in the genes of the circadian-clock components often affect flowering time (Nagel and Kay, 2012 [errata, 2013]). This is explained by the change of the timing of *CO* expression (Roden et al., 2002; Yanovsky and Kay, 2002). For example, *toc1-1*, a mutant of *TIMING OF CAB EXPRESSION 1* (*TOC1*) with a shorter circadian period, showed an early flowering phenotype under short-day (SD) conditions (Yanovsky and Kay, 2002). The shorter circadian period results in earlier (phase-advanced) *CO* expression, which causes the coincidence of high expression of *CO* and light even under SD conditions and consequently induces *FT* expression and flowering (Yanovsky and Kay, 2002). Roden et al. (2002) used non-24h light/dark cycles to alter the timing of circadian gene expression. They grew wild-type (WT) plants under light/dark cycles shorter or longer than 24 h comprised of a fixed light-to-dark ratio (in this case, 1 to 2) referred to as T-cycles, which caused later or earlier *CO* expression. Similar to the observations in the *toc-1-1* mutant, flowering was accelerated when *CO* was expressed in the light portion of the cycle. These results together show that the interaction between the circadian clock and light signal is the basis of daylength measurement in *Arabidopsis*. Similarly, it has been reported that several angiosperm species measure daylength through the interaction between the circadian clock and light signal (Song et al., 2010). Hence, it has been believed that this daylength measurement system based on the external and internal coincidence mechanisms is conserved in a wide range of plants. In contrast, in non-angiosperm plants, the mechanisms of daylength measurement are poorly understood.

To expand our knowledge of the mechanism of daylength measurement, we used an LD plant, *Marchantia polymorpha* s.sp. *ruderalis* (hereafter referred to as *M. polymorpha*). A model liverwort*, M. polymorpha* is suitable for genetic studies because of its low genetic redundancy of regulatory genes and various molecular biological techniques available (Ishizaki et al., 2016; Bowman et al., 2017; Kohchi et al., 2021). In *M. polymorpha*, growth-phase transition from vegetative to reproductive phase results in differentiation of gametangia and gametangiophores, Marchantiidae-specific stalked structures harboring gamatangia (Shimamura, 2016; Hisanaga et al., 2019; Yamaoka et al., 2021). Since liverworts represent one of the earliest diverged lineages of extant land plants (Puttick et al., 2018), studies with *M. polymorpha* have an additional merit of enhancing our understanding of the evolution of daylength-measurement mechanisms.

*M. polymorpha* lacks the *CO* ortholog and the counterpart of *FT* is also missing. But it possesses the single-copy ortholog or counterpart of *GI*, *FKF1*, and *CDF*, respectively (Kubota et al., 2014; Bowman et al., 2017; Linde et al., 2017). Recent studies demonstrated that the GI-FKF1 module is involved in the regulation of daylength-dependent growth-phase transition in *M. polymorpha*. Knockout of either Mp*FKF* or Mp*GI* suppressed the growth-phase transition irrespective of day-length conditions, while overexpression of either Mp*FKF* or Mp*GI* caused the growth-phase transition under non-inductive SD conditions (Kubota et al., 2014). Moreover, Mp*GI* showed a diel expression pattern similar to its *Arabidopsis* orthologue. These imply that, as in *Arabidopsis*, the circadian clock may be the key element in daylength measurement in *M. polymorpha*. Recent studies showed that *M. polymorpha* has a circadian oscillator that shares many components with *Arabidopsis* and there is the circadian regulation of nyctinastic thallus movement and photosynthesis (Linde et al., 2017; Lagercrantz et al., 2020; Cuitun-Coronado et al., 2022). However, it still remains to be examined whether the circadian clock is essential for daylength-dependent growth-phase transition in *M. polymorpha*.

In this study, we aimed to examine the involvement of the circadian clock in the regulation of daylength-dependent growth-phase transition through genetic (clock mutants), pharmacological (an inhibitor of a clock component), and physiological (non-24h light/dark cycles) approaches. All the results consistently indicate that the circadian clock is not the key element in daylength measurement of *M. polymorpha*. Moreover, our observations suggest that *M. polymorpha* responds to daylength based on the relative amount of light and darkness within a cycle. Our study provides new insights into evolution and diversification of daylength measurement systems in land plants.

## Materials and methods

### Plant materials and growth conditions

A male accession of *M. polymorpha*, Takaragaike-1 (Tak-1) was used as WT. Plants were grown on half-strength Gamborg’s B5 medium (Gamborg et al., 1968) containing 1% sucrose and 1.2% agar under 50–60 µmol photons m^−2^ s^−1^ continuous white fluorescent light at 22°C, unless otherwise specified (e.g. for non-24-h light/dark cycles). Mutants generated in this study by genome editing were named in accordance with the research community’s guidelines (Bowman et al., 2016).

### Induction of growth-phase transition

Gemmae were plated on half-strength Gamborg’s B5 medium containing 1% sucrose and 1.2% agar and kept in the dark for one day and then exposed to white fluorescent light with 50–60 μmol m^−2^ s^−1^ for an hour to synchronize germination. Then, gemmalings were transferred to non-inductive 8-hour light (L)/16-hour dark (D) (8L16D) conditions using 50–60 μmol m^−2^ s^−1^ white light supplemented with 45–50 μmol m^−2^ s^−1^ far-red light-emitting diodes (LED Profile Light for Plant, NAMOTO, Japan; peak emission at 730-735 nm). Ten days after growth under 8L16D conditions, they were transferred to various day-length conditions (see **Supplementary Figure 1**). The number of days required for the appearance of the first visible gametangiophore primordium after the transfer to various day-length conditions was scored.

### Construction of plasmids

The genomic sequence of the Mp*PSEUDO-RESPONSE REGULATOR* (Mp*PRR*) promoter region (2974 bp) was amplified from Tak-1 genomic DNA using primers: 5′-ATCCGGTACCGAATTCATAAGACGTTTGATGTGGCAATGG-3′ and 5′-GTGCGGCCGCGAATTCATCAGAAGAGAAATTGCAGTACG-3′. The amplified fragment was cloned into the *Eco*RI site of pENTR1A vector (Thermo Fisher Scientific). The inserted fragments were then transferred into pMpGWB331 (Ishizaki et al., 2015) to generate *_pro_* Mp*PRR* : *LUC* plasmid.

To generate mutants for Mp*TOC* and Mp*PRR*, oligonucleotides: MpTOC_sgRNA1, 5′-CTCGTCAACCTCGCCGAAGTGTCA-3′ and 5′-AAACTGACACTTCGGCGAGGTTGA-3′, MpTOC_sgRNA2, 5′- CTCGACACACAAGGAAACCCTGCT-3′ and 5′- AAACAGCAGGGTTTCCTTGTGTGT-3′, MpPRR_sgRNA1, 5′- CTCGTGCAGAGTCCAGTGAGAAGG-3′ and 5′- AAACCCTTCTCACTGGACTCTGCA-3′, MpPRR_sgRNA2, 5′- CTCGAGCGGACTTAACGTGAGAGG-3′ and 5′- AAACCCTCTCACGTTAAGTCCGCT-3′ were used as guide RNA (gRNA) sequences for CRISPR/Cas9-mediated genome editing. The gRNAs were subcloned into the *Bsa*I site of pMpGE_En04. The resultant constructs were transferred to the pMpGE010 vector by the Gateway LR reaction as described previously (Sugano et al., 2018).

### Plant transformation

Transformation of *M. polymorpha* (Tak-1 accession) was performed via the AgarTrap method as described previously (Tsuboyama and Kodama, 2014). Transformants were selected on the medium supplemented with 100 μg ml^−1^ cefotaxime and either 10 μg ml^−1^ hygromycin or 0.5 μM chlorsulfuron depending on the selection marker genes in the binary vectors and cultivated for about 1 month to obtain G1 gemmae (Ishizaki et al., 2016).

### Bioluminescence monitoring

Gemmalings were grown on the medium at 22°C under constant light conditions for 7-10 days. Then, the plants were transferred to 12L12D conditions. For experiments examining the effect of AMI-331 (Tokyo Chemical Industry, product No. A3352) treatment on the circadian period, the plants were transferred to the medium containing various concentration of AMI-331 at the same time. 1-2 days after the transfer, plants were sprayed with a 1 mM solution of D-Luciferin (Biosynth) prepared in 0.005% (v/v) Triton X-100 (Sigma-Aldrich). After culturing the plants in 12L12D conditions for additional 2 days, bioluminescence monitoring was performed as described previously (Muranaka et al., 2015). A time-series analysis was performed using R 3.5.0 (https://www.r-project.org/). The free-running priods (FRPs) and relative amplitude errors (RAEs) were estimated using the fast Fourier transform nonlinear least squares (FFT-NLLS) method as described previously (Muranaka and Oyama, 2016).

## Results

### *M. polymorpha* is an obligate long-day plant and requires multiple long days

It has long been known that reproductive transition in *M. polymorpha* requires LD conditions (Wann, 1925), and recent studies demonstrated the importance of far-red light, which is abundant in sunlight but is insufficient in fluorescent lamps, in daylength response (Chiyoda et al., 2008; Kubota et al., 2014; Inoue et al., 2019). However, physiological details and molecular basis of day-length response remain to be explored.

**Figure 1:**
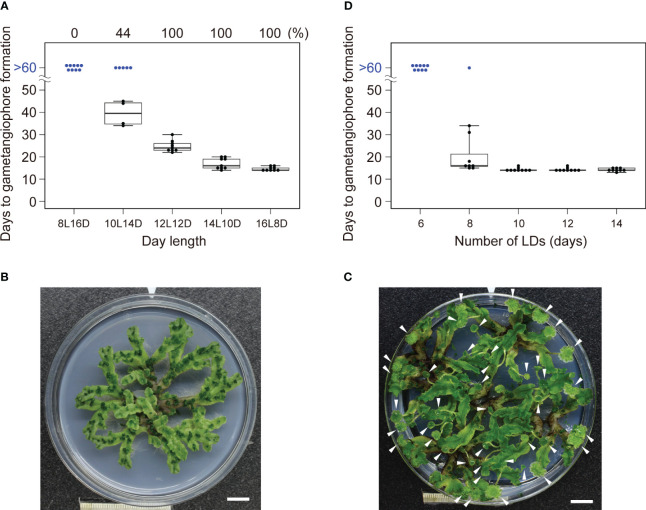
Effects of daylength on the induction of gametagiophore formation. **(A)** Timing of gametangiophore formation in WT under various daylength conditions. 10-day-old gemmalings grown under 8L16D conditions were transferred to various daylength conditions. Number of days required for the formation of the first visible gametangiophore primordium were quantified and presented as a boxplot (n = 9). Blue dots at >60 days represent plants with no gametangiophore primordia on day 60 after the transfer. Percentage of plants with gametangiophore primordia is given above the graph. **(B)** A 100-day-old WT plant grown under 8L16D conditions. Scale bar = 1 cm. **(C)** 30-day-old WT plants grown under 16L8D conditions. Scale bar = 1 cm. Arrowheads indicate developing gametangiophores. **(D)** Effect of the number of the LD on reproductive transition. 10-day-old gemmalings grown under 8L16D conditions were transferred to 16L8D. After being subjected to 16L8D conditions for designated days, plants were returned to 8L16D conditions. Number of days required for the formation of the first visible gametangiophore primordium from the start of the 16L8D treatment were quantified and presented as a boxplot (n=9). Blue dots at >60 days represent plants with no gametangiophore primordia on day 60 after the transfer back to 8L16D.

To examine whether *M. polymorpha* has a critical daylength for reproductive transition, WT plants of a widely used accession, Takaragaike-1 (Tak-1), were grown under various daylengths from 8-h light to 16-h light in 24-h light/dark cycles (*i. e.*, 8L16D to 16L8D), and the number of days required for the appearance of the first visible gametangiophore primordium, a convenient indicator of reproductive transition, was examined under dissecting microscope. As previously reported, Tak-1 WT plants formed the first visible gametangiophore primordium within 14 days under 16L8D conditions, and none under 8L16D conditions after 60 days (**Figure 1A**; Kubota et al., 2014). Under 8L16D conditions, plants remained vegetative for more than 100 days (**Figure 1B**, see **Figure 1C** for plants with gametangiophores grown under 16L8D), suggesting that *M. polymorpha* is an obligate LD plant, in which daylength longer than a threshold is absolutely required for the induction of growth-phase transition. Under 10L14D conditions, 56% of the plants did not form any gametangiophores after 60 days, whereas all plants formed gametangiophores under 12L12D conditions (**Figure 1A**), indicating that the critical daylength (defined here as the daylength at which 50% of plants undergo reproductive transition; see Thomas and Vince-Prue, 1997) of Tak-1 WT accession is about 10 hours.

We next examined whether a single LD is sufficient for reproductive transition or multiple LDs are required. *Arabidopsis* plants of accession Col-0 requires only 1 day of LD for induction of flowering (Corbesier et al., 1996). By contrast, Tak-1 WT plants subjected to LD (16L8D) conditions for less than 6 days did not form any gametangiophores after 60 days, 8 or more days of LD were required for the induction of gametangiophore formation (**Figure 1D**). These differences suggest that there is the difference in the sensitivity to LD and/or in the nature of inductive signal(s) generated under LD conditions between the two species.

### Alteration of circadian rhythms did not change perception of daylength

**Figure 2:**
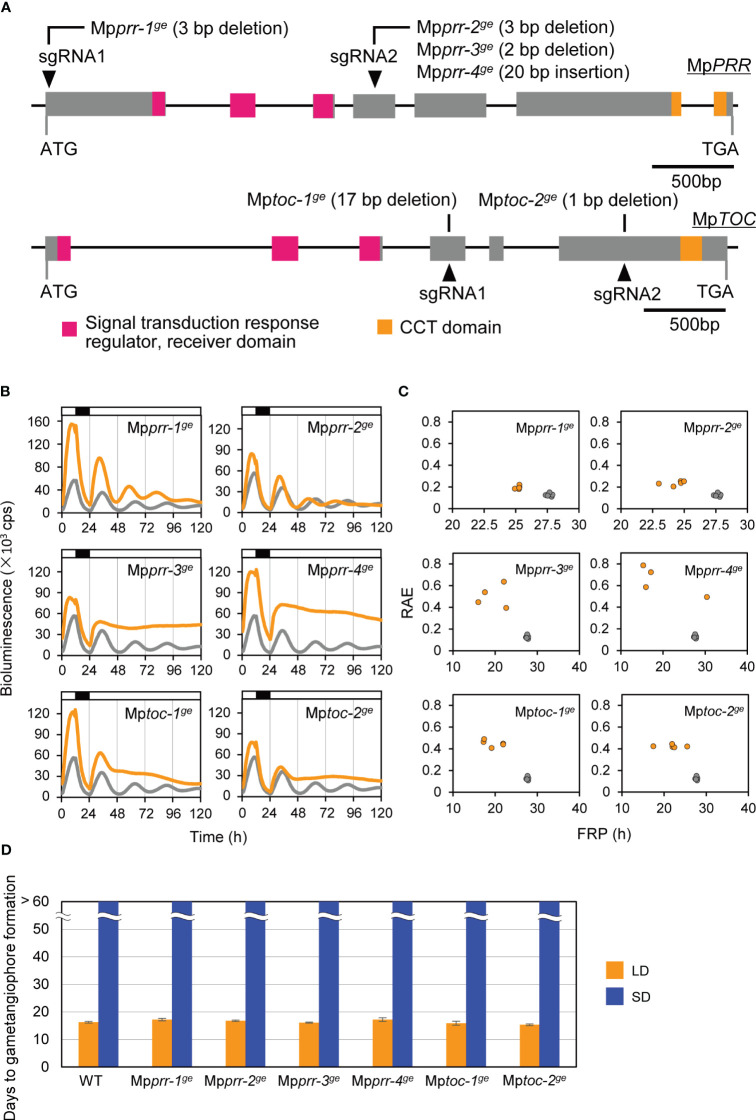
Effects of clock mutations on bioluminescence rhythms and the timing of the growth-phase transition. **(A)** Mp*PRR* and Mp*TOC* gene structure and positions of Mp*prr* and Mp*toc* mutations. Gray boxes represent coding regions. Regions corresponding to conserved domains are shown in color. Arrowheads represent the positions of gRNAs. See **Supplementary Figure 1** for details of Mp*prr* and Mp*toc* mutations. **(B, C)** Bioluminescence rhythms of Mp*PRR pro*:LUC in clock mutants (orange) and WT (gray). **(B)** Bioluminescence of Mp*PRR pro*:LUC was recorded under 12L12D conditions and constant‐light (LL) conditions in clock mutants and WT. White and black bars above the graph indicate light and dark periods, respectively. The representative of five replicates is shown. **(C)** Relative amplitude error (RAE) and free-running period (FRP) length were estimated by FFT-NLLS using data collected from 36 h to 120 h from the start of the monitoring. Each plotted dot represents an independent line. **(D)** Timing of gametangiophore formation in clock mutants and WT. 10-day-old gemmalings grown under 8L16D conditions were transferred to 16L8D (LD, orange) or 8L16D (SD, blue) conditions. Number of days required for the formation of the first visible gametangiophore primordium were quantified. Bars represent mean ± SEM (n = 9). See **Supplementary Figure 3** for photographs of representative plants grown in 8L16D conditions.

To examine whether the circadian clock is a key component in daylength measurement of *M. polymorpha*, we first generated clock mutants with various circadian-rhythm phenotypes and examined their daylength response. A CRISPR/Cas9 system was used with two different gRNAs to generate mutations in Mp*PRR* or Mp*TOC1* in a genetic background carrying a luciferase reporter under the control of Mp*PRR* promoter (*_pro_* Mp*PRR* : *LUC*) which allows bioluminescence-based rhythm assays. As previously reported, the parental *_pro_* Mp*PRR* : *LUC* lines showed diurnal expression patterns under constant-light (LL) conditions for several days (**Figure 2B**; Linde et al., 2017). We obtained four independent Mp*prr^ge^* lines and two independent Mp*toc^ge^* lines showing altered circadian-rhythm phenotypes (**Figure 2A**; **Supplementary Figure 2**). Two Mp*prr^ge^* lines, Mp*prr-1^ge^* and Mp*prr-2^ge^*, showed short-period phenotypes under LL conditions (25.3 h versus 27.9 h in WT). The rest of four lines, Mp*prr-3^ge^*, Mp*prr-4^ge^*, Mp*toc-1^ge^* and Mp*toc-2^ge^* showed arrhythmic phenotypes under LL conditions (**Figures 2B, C**). We then compared the timing of gametangiophore formation between WT and these 6 clock mutants under LD (16L8D) and SD (8L16D) conditions. Interestingly, there were no significant differences in the timing of gametangiophore formation between WT and clock mutants under both daylength conditions (**Figure 2D**; **Supplementary Figure 3**). In contrast to the previous observation that phase-advanced *toc1-1* mutant in *Arabidopsis* shows early flowering under SD conditions (Yanovsky and Kay, 2002), neither of the short-period mutants (Mp*prr-1^ge^* and Mp*prr-2^ge^*) formed gametangiophore under SD conditions.

**Figure 3:**
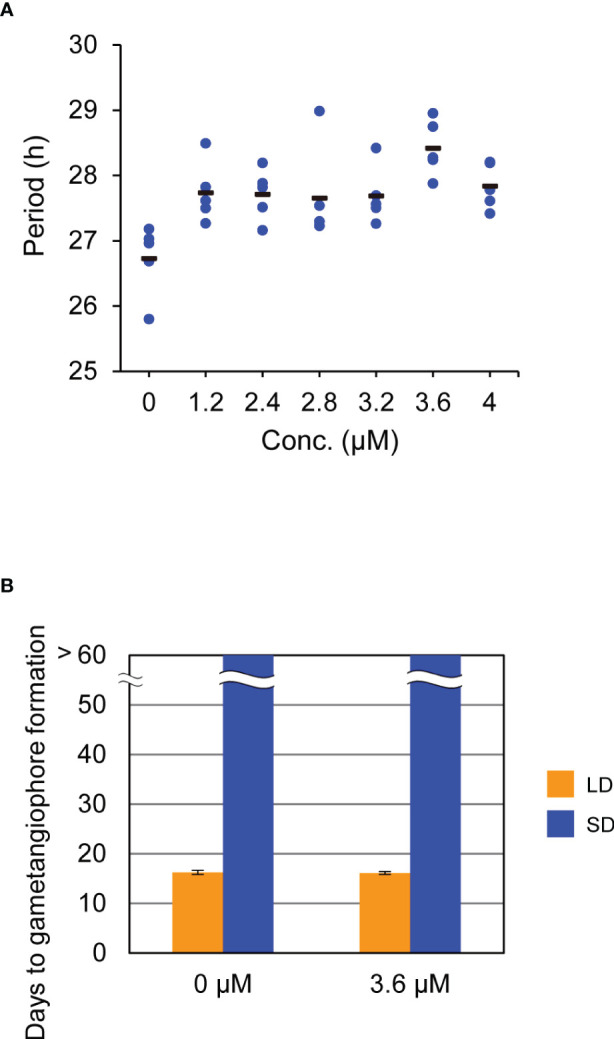
Effects of AMI-331 treatment on bioluminescence rhythms and the timing of the growth-phase transition. **(A)** Effects of AMI-331 treatment on period length of bioluminescence rhythms of Mp*PRR pro*:LUC under constant light (LL) conditions. Each plotted dot represents an individual of five biological replicates. The bars represent the averages of them. Period length was estimated by FFT-NLLS using data collected from 12 h to 120 h after transfer to LL from 12L12D conditions. **(B)** Effects of AMI-331 treatment on the timing of gametangiophore formation. 10-day-old gemmalings grown under 8L16D conditions on the medium were transferred to medium containing 3.6mM AMI-331 or AMI 331-free medium. Subsequently, they were grown under 16L8D (LD, orange) or 8L16D (SD, blue) conditions. Number of days required for the formation of the first visible gametangiophore primordium were quantified. Bars represent mean ± SEM (n = 8). See **Supplementary Figure 4** for photographs of representative plants grown in 8L16D conditions.

To further investigate the role of the circadian clock in daylength measurement, we attempted to use the pharmacological approach (Nakamichi et al., 2022). Recent study showed that a chemical called AMI-331, an inhibitor of casein kinase 1 (CK1), lengthens circadian period by inhibiting the phosphorylation of TOC1 and PRR5 in *Arabidopsis* (Uehara et al., 2019; Saito et al., 2019). Because a single-copy gene (Mp6g17600) for CK1, with more than 90% identity with Arabidopsis CK1 in the deduced amino-acid sequences of the kinase domain, and the ortholog or counterpart of *TOC1* and *PRR5* (mentioned above) are present in *M. polymorpha* genome, we expected that AMI-331 is also effective in lengthening the circadian period in *M. polymorpha*. Bioluminescence rhythms of *_pro_* Mp*PRR* : *LUC* under LL conditions were monitored for plants grown on the growth medium supplemented with AMI-331 at various concentrations. Although its effect was much smaller than that observed in *Arabidopsis* (Saito et al., 2019), AMI-331 treatment lengthened the circadian period in *M. polymorpha* as well (**Figure 3A**). The most effective concentration for lengthening the circadian period was 3.6 µM with a 2-h period lengthening (**Figure 3A**). Based on this observation, we treated WT plants with 3.6 µM AMI-331 and investigated its effect on the timing of gametangiophore formation under LD (16L8D) and SD (8L16D) conditions. Consistent with the results of clock mutants, lengthening circadian period by AMI-331 treatment had no significant effects on the timing of gametangiophore formation (**Figure 3B**; **Supplementary Figure 4**). These results taken together indicate that, in contrast to *Arabidopsis*, the circadian clock is not an essential component in daylength measurement in *M. polymorpha*.

### Relative duration of light and darkness within a cycle may determine daylength perception

**Figure 4:**
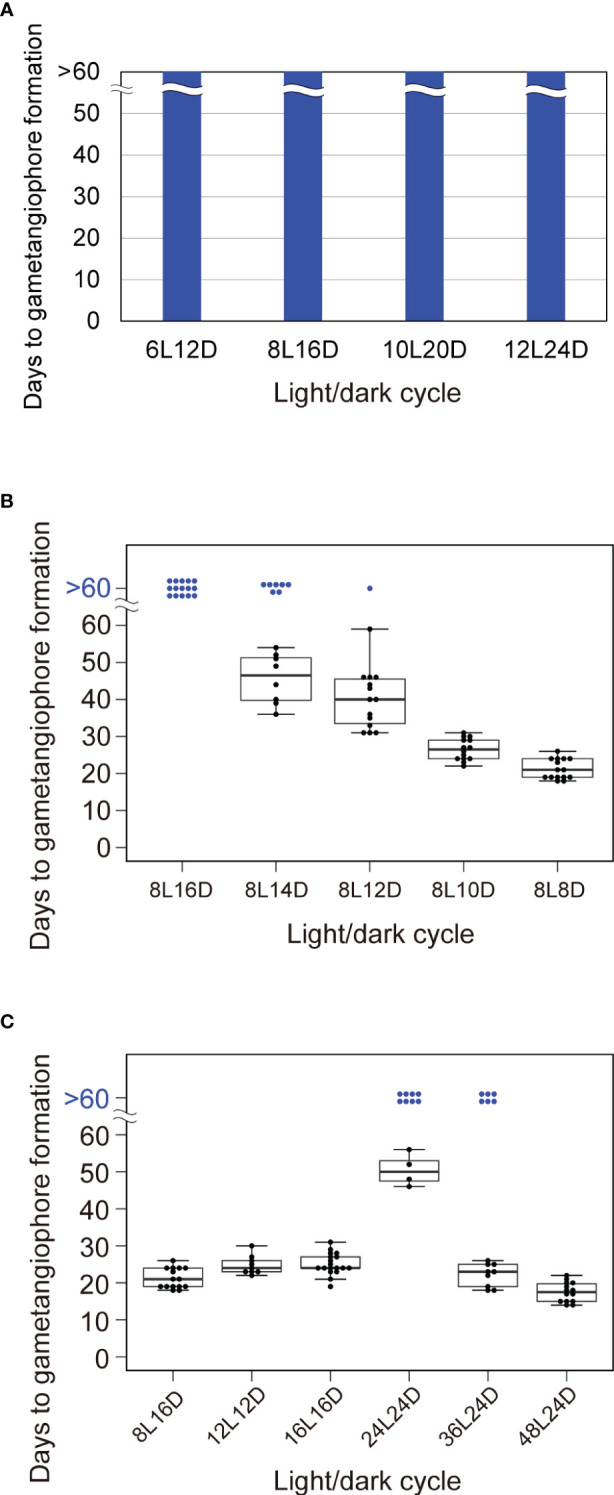
Effects of non-24-h light/dark cycles on circadian gene expression and the timing of growth-phase transition. **(A)** Timing of gametangiophore formation in WT plants under T-cycles varying in duration from 18 to 36 h, comprised of light and dark periods in a ratio of 1 to 2. 10-day-old gemmalings grown under 8L16D conditions were transferred to designated T-cycles. Number of astronomical days (i.e. in 24-h day) required for the formation of the first visible gametangiophore primordium were quantified (n = 15). See **Supplementary Figure 5** for photographs of representative plants. **(B)** Timing of gametangiophore formation in WT plants under NH-cycles varying in duration from 16 to 24 h, comprised of a fixed light period of 8 h and a dark period of variable lengths. 10-day-old gemmalings grown under 8L16D conditions were transferred to NH-cycles. Number of astronomical days required for the formation of the first visible gametangiophore primordium were quantified and presented as a boxplot (n = 15). Blue dots at “>60 days” represent plants with no gametangiophore primordia on day 60 (of 24-h day) in respective light/dark cycles. See **Supplementary Figure 5** for photographs of representative plants from a similar but separate set of experiments. **(C)** Correlation between light/dark ratios and the timing of growth-phase transition is limited within a certain duration of a cycle. 10-day-old gemmalings grown under 8L16D conditions were transferred to 8L8D, 12L12D, 16L16D, 24L24D, 36L24D, and 48L24D conditions. Number of astronomical days required for the formation of the first visible gametangiophore primordium were quantified and presented as a boxplot (n ≥ 9). Blue dots at “>60 days” represent plants with no gametangiophore primordia on day 60 (of 24-h day) in respective light/dark cycles. See **Supplementary Figure 5** for photographs of representative plants grown in 16L16D, 24L24D, and 36L24D conditions. Representative plants grown in 8L8D conditions in a separate set of experiments are also shown in **Supplementary Figure 5**.

To further confirm that the circadian clock is not a key component in daylength measurement, we performed experiments involving non-24-h light/dark cycles of various cycle period lengths (T-cycles). In general, longer T cycles advance the timing of circadian gene expression, whereas shorter T cycles delay it (Aschoff, 1960). Based on this, T-cycle experiments have been used to demonstrate the involvement of the circadian clock in daylength measurement (Roden et al., 2002; Yanovsky and Kay, 2002 for *Arabidopsis*). Using T cycles described earlier in the analysis with *Arabidopsis* (Roden et al., 2002), we subjected Tak-1 WT plants to T cycles ranging from 18 to 36 h comprised of light and dark periods at the ratio of 1 to 2; T = 18 h (6L12D), 24 h (8L16D), 30 h (10L20D) and 36 h (12L24D). We then analyzed the effects of T-cycles on the timing of gametangiophore formation. WT plants did not develop gemetangiophores in >60 days under any T-cycle conditions (**Figure 4A**; **Supplementary Figures 5A–C**). This is in contrast with the case of *Arabidopsis* in which plants showed accelerated flowering under T-cycles longer than 24 h (*i.e.* 28, 30, and 32 h). Taken together with the results with clock mutants, the notion that *M. polymorpha* has a daylength measurement system in which circadian clock does not play an essential role is supported. Additional comparisons showed that daylength measurement in *M. polymorpha* does not simply rely on the duration of either light or darkness, as light/dark cycles containing the same light periods (12L12D and 12L24D) or dark periods (6L12D and 12L12D) have different effects on the induction of gametangiophore formation (**Figures 1A**, **4A**).

Because all T-cycles comprised of light and dark periods in a ratio of 1 to 2 had essentially the same effects on the induction of gametangiophore formation, although the total duration of a cycle differs considerably from 18 to 36 h (**Figure 4A**), we hypothesized that daylength measurement in *M. polymorpha* is based on the relative duration of light and darkness within a cycle. To test the hypothesis, we next applied Nanda-Hamner (NH) cycles comprised of a constant light period altering with a dark period of variable lengths (Teets and Meuti, 2021). If our hypothesis is correct, increasing or decreasing the hours of darkness should either inhibit or accelerate the induction of gametangiophore formation. Here we set light periods to 8 h and exposed WT plants to the following NH-cycle conditions; NH = 24 h (8L16D), 22 h (8L14D), 20 h (8L12D), 18h (8L10D) and 16h (8L8D). Although 24-h NH cycles (i.e., normal 24-h short days) were perceived as non-inductive daylength conditions, 53% of the plants formed gametangiophores under 22-h NH cycles and days to gametangiophore formation decreased as the length of dark period were further shortened (**Figure 4B**; **Supplementary Figures 5D, E**). These observations support the hypothesis that daylength perception of *M. polymorpha* is based on the relative duration of light and darkness within a cycle.

Additional light/dark cycles were also tested (**Figure 4C**). To examine whether the same conclusion holds for cycles comprised of a constant dark period altering with a light period of various lengths, we compared the timing of gametangiophore formation between 8L16D (normal 24-h short days) and 16L16D conditions. Consistent with the observation that decrease of the proportion of dark period accelerated the induction of gametangiophore formation (**Figure 4B**), increase of the proportion of light period from 33% (8L16D) to 50% (16L16D) has an accelerating effect (**Figures 4B, C**). Notably, gametangiophore formation was induced at almost the same timing under 12L12D and 16L16D conditions (24.8 and 25.1 days, respectively), both of which have a light-to-dark ratio of 1 to 1 (**Figure 4C**). Lastly, we investigated whether the correlation between light/dark ratios and the timing of induction is observed under cycles of extremely long durations as well. Unexpectedly, effects of 24L24D conditions on the timing of induction were critically different from those of 12L12D and 16L16D conditions (**Figure 4C**; **Supplementary Figures 5F–H**). Under 24L24D conditions, 66% of the plants did not form any gametangiophores after 60 days (**Figure 4C**). This implies that the correlation between light/dark ratios and the timing of induction breaks down around a certain threshold duration. At the same time, the results raise the possibility that cycles comprised of dark periods of 24 h or longer are perceived as non-inductive conditions even when combined with sufficiently long light periods. To test this possibility, we observed the timing of gametangiophore formation under 36L24D and 48L24D conditions. Under 36L24D conditions, 40% of the plants did not form any gemetangiophores after 60 days. However, days to gametangiophore formation of the induced plants were decreased compared to 24L24D (50.5 days in 24L24D vs 22.1 days in 36L24D conditions) (**Figure 4C**; **Supplementary Figures 5I, J**). Under 48L24D conditions, all the plants formed gametangiophores at earlier dates (**Figure 4C**). These results indicate that increase of the proportion of light is effective to accelerate the induction of gametangiophore formation even under cycles with extremely long durations of darkness.

## Discussion

In the present study, we showed that alterations of circadian rhythms by genetic and pharmacological approaches have no significant effects on the timing of growth-phase transition in an obligate long-day plant *M. polymorpha* (accession Tak-1). Additionally, non-24h light/dark-cycle experiments suggest that there is the correlation between light-to-dark ratios in a cycle and the timing of growth-phase transition. These results taken together indicate that circadian clock is not an essential component in the daylength measurement of *M. polymorpha*. This is in contrast to angiosperms in which circadian clock plays a pivotal role. It is likely that daylength measurement in *M. polymorpha*, instead, is dependent on the light-to-dark ratio within a cycle. Inductive signal, which is somehow diminished in the dark, may be generated during light periods through phytochrome far-red high-irradiance response (Inoue et al., 2019). Given that *M. polymorpha* (accession Tak-1) requires multiple LDs (**Figure 1D**), presumptive inductive signal may accumulate during repeated LDs to reach a certain threshold required for growth phase transition. Hence the balance between light and dark periods will affect whether the inductive signal accumulates and/or whether the level of the accumulated inductive signal exceeds the threshold. Search for such a signal is an important project for future study.

One potential explanation for the difference of the daylength measurement may be related to the robustness of circadian oscillations. Gene numbers and complexity have generally increased during evolution of plant circadian clocks (Linde et al., 2017), and complexity of the oscillators is considered to bring robustness to environmental perturbation (Shalit-Kaneh et al., 2018). The circadian clock of *M. polymorpha* has fewer components than *Arabidopsis* and has been considered to be less robust (Linde et al., 2017). Linde et al. (2017) showed that clock gene expression immediately adapts to 6L6D conditions after transfer of plants from 12L12D conditions, which suggests that the underlying circadian clock regulation on gene expression is masked by the effect of light/dark cycles. Due to this property, the effect of the circadian clock may be overridden by the effect of light/dark cycles in daylength measurement of *M. polymorpha*. Whether circadian clocks of non-angiosperm plants with smaller number of components are less robust and hence they do not play a central role on daylength measurement awaits further study.

To lengthen circadian period of *M. polymorpha*, we used AMI-331, a period-lengthening chemical reported in *Arabidopsis* (Saito et al., 2019). In *Arabidopsis*, AMI-331 inhibits the activity of CK1-family proteins, and increases PRR5 and TOC1 protein amounts (Uehara et al., 2019; Saito et al., 2019). In this study, we showed that AMI-331 treatment also lengthens circadian period of *M. polymorpha* (**Figure 3A**), suggesting that AMI-331 inhibits the activity of CK1 encoded by a single-copy gene (Mp6g17600), affecting MpPRR and MpTOC1 protein levels, and therby resulting in lengthening of circadian period. Further analysis of the role of CK1 in the *M. polymorpha* circadian clock could provide a clue to understand the structure of the *M. polymorpha* circadian oscillator and the evolution of the land plant circadian clocks.

In addition to contrasting difference in the involvement of the circadian clock in daylength measurement, *Arabidopsis* and *M. polymorpha* differ in their sensitivity to daylength. *Arabidopsis* (accession Col-0) requires only 1 day of LD for reproductive transition, whereas *M. polymorpha* (accession Tak-1) requires 8 or more LDs. Similar to *M. polymorpha*, an SD plant *Chrysanthemum morifolium* requires repeated SDs for inflorescence development (Adams et al., 1998). In *Chrysanthemum, CsFTL3* (*FLOWERING LOCUS T-like 3*) expression gradually increases during repeated SDs, and inflorescence development is induced when CsFTL3 signal reaches the threshold (Nakano et al., 2013). Recently, it was shown that Cs*FLT3* expression is positively regulated during SDs by a feedback mechanism involving the complex of CsFTL3 and its partner CsFDL1 (FD-like1) (Nakano et al., 2019). Exploration of genes whose expression gradually change during repeated LDs and possible epigenetic changes responsible for it will provide insights into the mechanisms of daylength measurement in *M. polymorpha*.

We showed that the circadian clock does not have the central role in daylength measurement of *M. polymorpha*. Further studies are required to understand how circadian clock-regulated GI-FKF1 module and a possible downstream factor CDF are involved in daylength response of *M. polymorpha*. Recent progress has uncovered key steps in sexual reproduction in *M. polymorpha* (Hisanaga et al., 2019; Yamaoka et al., 2021). The earliest step, gametangium primordia initiation, is regulated by a basic helix-loop-helix (bHLH) gene *BONOBO* (*BNB*), whose expression is induced by LD, and another bHLH gene *LOTUS JAPONICUS ROOTHAIRLESS LIKE* (*LRL*) whose protein forms a heterodimer with BNB (Yamaoka et al., 2018; Saito et al., 2023). A conserved regulatory module of microRNA156/529 and squamosa promoter-binding-protein-like (SPL) is likely to be involved in daylength response of *M. polymorpha* (Yamaoka et al., 2021). A counterpart of angiosperm miR156, miR529c, prevents *M. polymorpha* from reproductive transition in non-inductive SD conditions by restricting Mp*SPL2* activity (Tsuzuki et al., 2019). Other factors acting possibly downstream of daylength measurements have also been identified, such as Mp*DELLA* and Mp*PIF* (Hernández-García et al., 2021) and Mp*KANADI* (Briginshaw et al., 2022). It is likely that these factors control daylength-dependent reproductive transition through regulation of *BNB* expression. A recent work reports that a signaling pathway of unidentified gibberellin-related compounds is actually involved in *BNB* expression under LD condition (Sun et al., 2023). The rapidly accumulating knowledge will help us to uncover the molecular mechanism underlying daylength measurement and subsequent pathway(s) leading to *BNB* expression. Further analysis at the molecular level in *M. polymorpha* should provide important insights into understanding evolution and diversification of daylength measurement systems among land plants.

## Data availability statement

The raw data supporting the conclusions of this article will be made available by the authors, without undue reservation.

## Author contributions

YK: Conceptualization, Funding acquisition, Investigation, Writing – original draft. KI: Conceptualization, Funding acquisition, Investigation, Writing – review & editing. YT: Writing – review & editing, Supervision. SY: Supervision, Writing – review & editing, Funding acquisition. TA: Funding acquisition, Supervision, Writing – review & editing, Conceptualization.
