# Supplementary material for: Circadian clock does not play an essential role in daylength measurement for growth-phase transition in Marchantia polymorpha
Source: Front Plant Sci. 2023 Nov 8;14:1275503. doi: 10.3389/fpls.2023.1275503 (PMC10673691; doi:10.3389/fpls.2023.1275503)
Supplement: Supplementary file 1 [file DataSheet_1.docx]

Supplementary Material

Circadian clock does not play an essential role in daylength measurement for growth-phase transition in *Marchantia polymorpha*.

Yuki Kanesaka^1^, Keisuke Inoue^1,2^, Yuki Tomita^1^, Shohei Yamaoka^1^, Takashi Araki^1*^

**^1^** Graduate School of Biostudies, Kyoto University, Sakyo-ku, Kyoto 606-8501, Japan

**^2^** Center for Living Systems Information Science, Graduate School of Biostudies, Kyoto University, Sakyo-ku, Kyoto 606-8501, Japan

*** Correspondence:** Takashi Araki: [araki.takashi.3a@kyoto-u.ac.jp](mailto:araki.takashi.3a@kyoto-u.ac.jp)


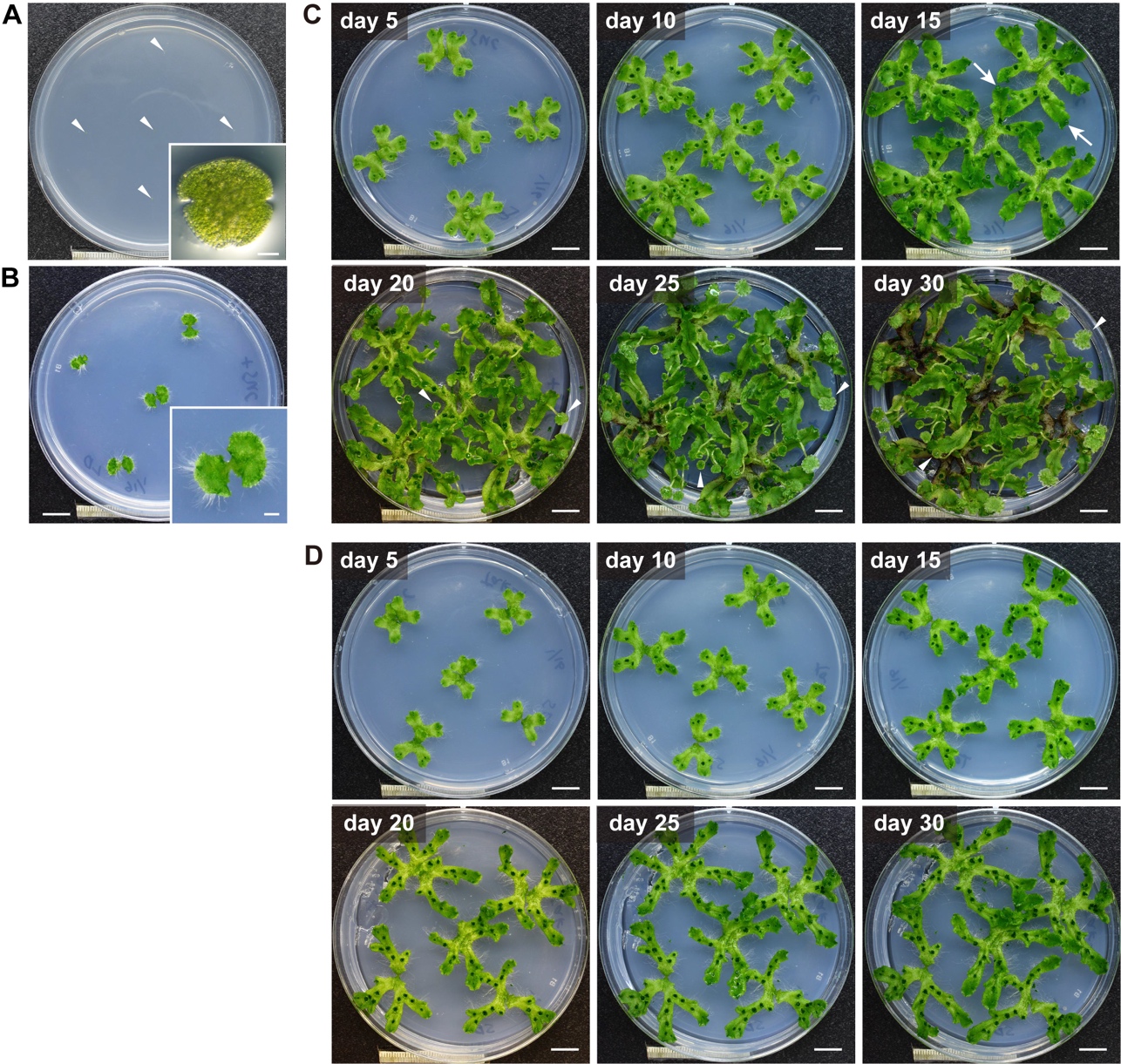
­­­

**Supplementary Figure 1.** **Growth of *M. polymorpha* plants in experimental conditions for testing daylength response.**

(A) Fresh gemmae placed on a 9-cm plate before placing in the dark for 1 day. Arrowheads indicate the plated gemmae. Magnified image of a gemma on the plate is shown in inset. (B) Typical plants after 10 days of growth in the non-inductive light condition. Magnified image of the plant is shown in inset. In this particular case, continuous white-light condition without supplement of far-red light, instead of SD (8L16D) conditions with supplement of far-red light, was used as a non-inductive light condition because of slightly better growth. These plants are transferred to various light/dark-cycle conditions. (C) Typical images of growth of plants after transfer to an inductive LD (16L8D) condition supplemented with far-red light at the indicated dates. Arrowheads and arrows indicate the representatives of gametangiophores and their primordia, respectively. (D) Typical images of growth of plants after transfer to a non-inductive SD (8L16D) condition supplemented with far-red light at the indicated dates. Dark green spots on the thallus are gemma cups filled with gemmae. Scale bar = 1 cm (A to D), 100 µm (inset in (A)), 2 mm (inset in (B)).


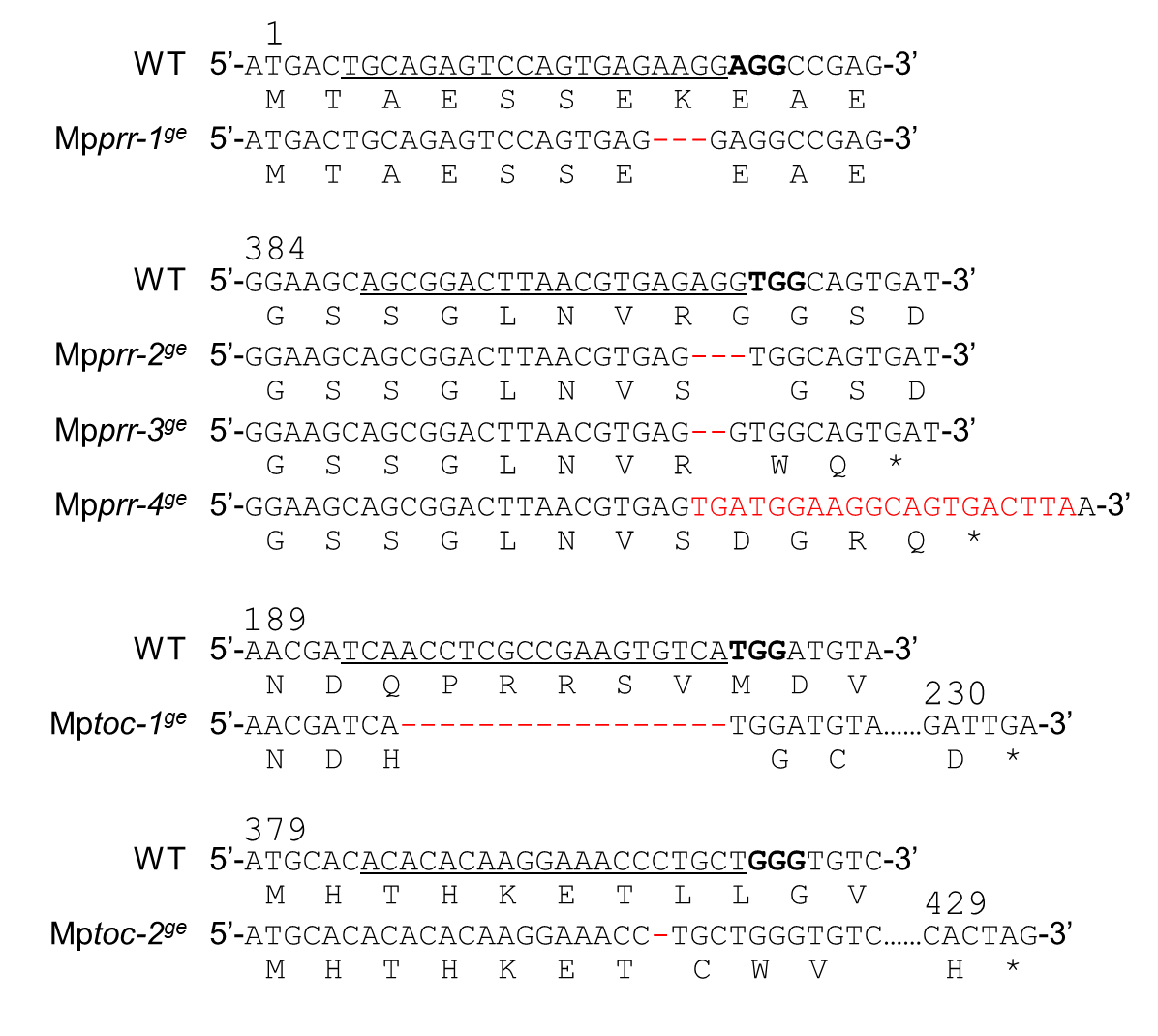


**Supplementary Figure 2.** **Mutations in Mp*PRR* and Mp*TOC*.**

Sequence alignments of WT and mutant alleles of Mp*PRR* and Mp*TOC*. A part of nucleotide and corresponding amino acid sequences are depicted. The sequence for guide RNA (gRNA) used for genome editing is underlined. The protospacer adjacent motif (PAM) sequence for CRISPR/Cas9 is shown in bold. Deletions and insertions are shown in red.


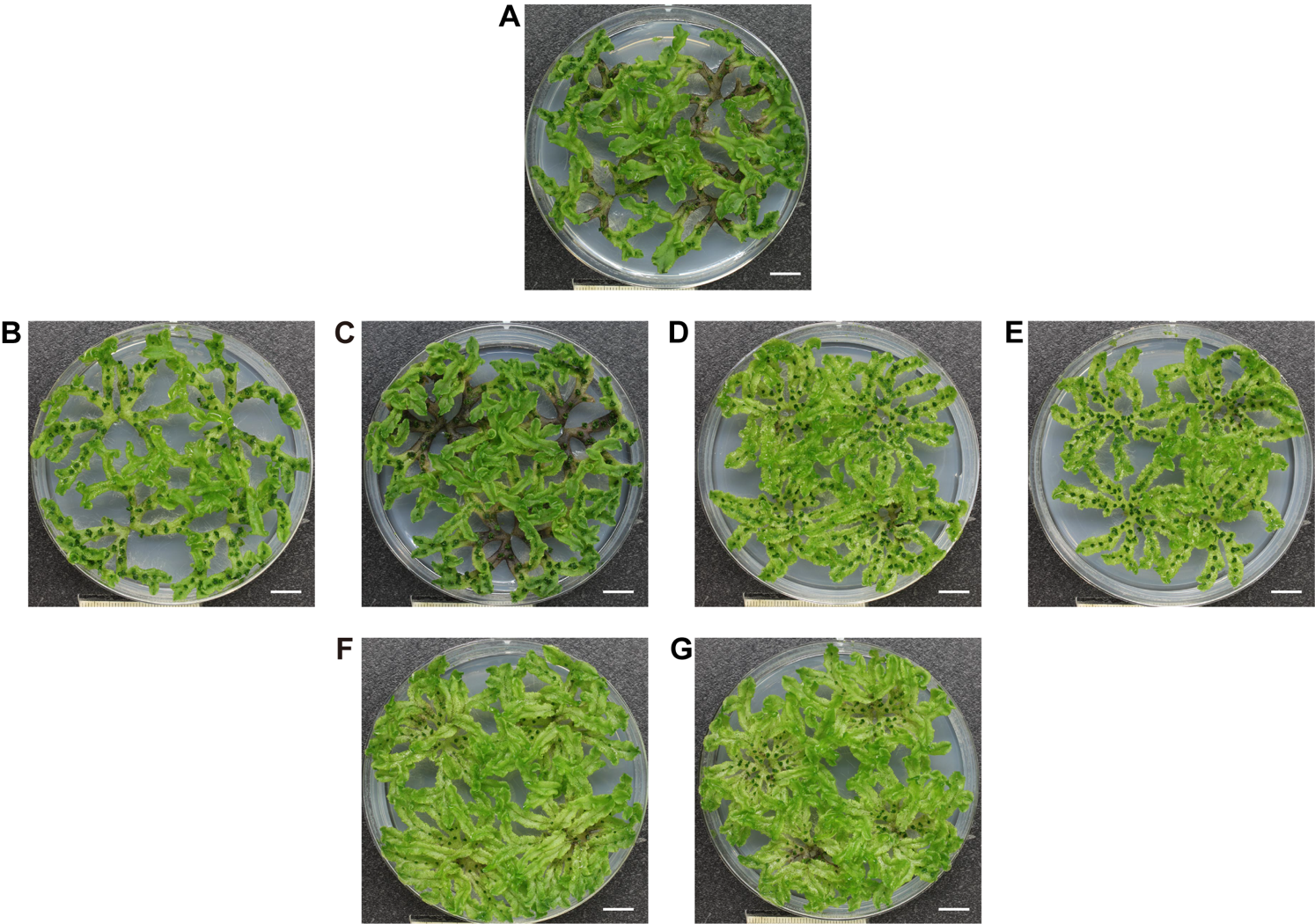


­­­

**Supplementary Figure 3.** **Representative plants of Mp*prr* and Mp*toc1* mutants.**

Images of representative plants grown for about 60 days under SD (8L16D) conditions (**Fig. 2D**). (A) WT (*_pro_*Mp*PRR*:*LUC*), (B) Mp*prr-1^ge^* (short period), (C) Mp*prr-2^ge^* (short period), (D) Mp*prr-3^ge^* (arrhythmic), (E) Mp*prr-4^ge^* (arrhythmic), (F) Mp*toc-1^ge^* (arrhythmic), and (G) Mp*toc-2^ge^* (arrhythmic). None of them developed gametangiophores. Dark green spots on the thallus are gemma cups filled with gemmae. Scale bar = 1 cm.


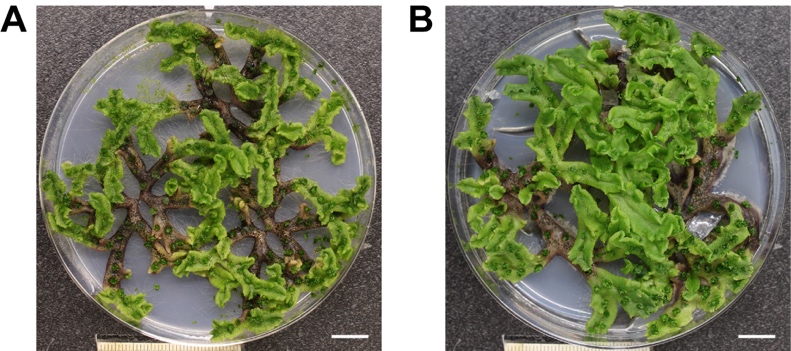


**Supplementary Figure 4.** **Representative plants grown in the presence of AMI-331.**

Images of representative plants grown for about 60 days under SD (8L16D) conditions with or without (**Fig. 3B**). (A) Plants grown on the medium without AMI-331. (B) Plants grown on the medium with 3.6 µM AMI-331. Scale bar = 1 cm.


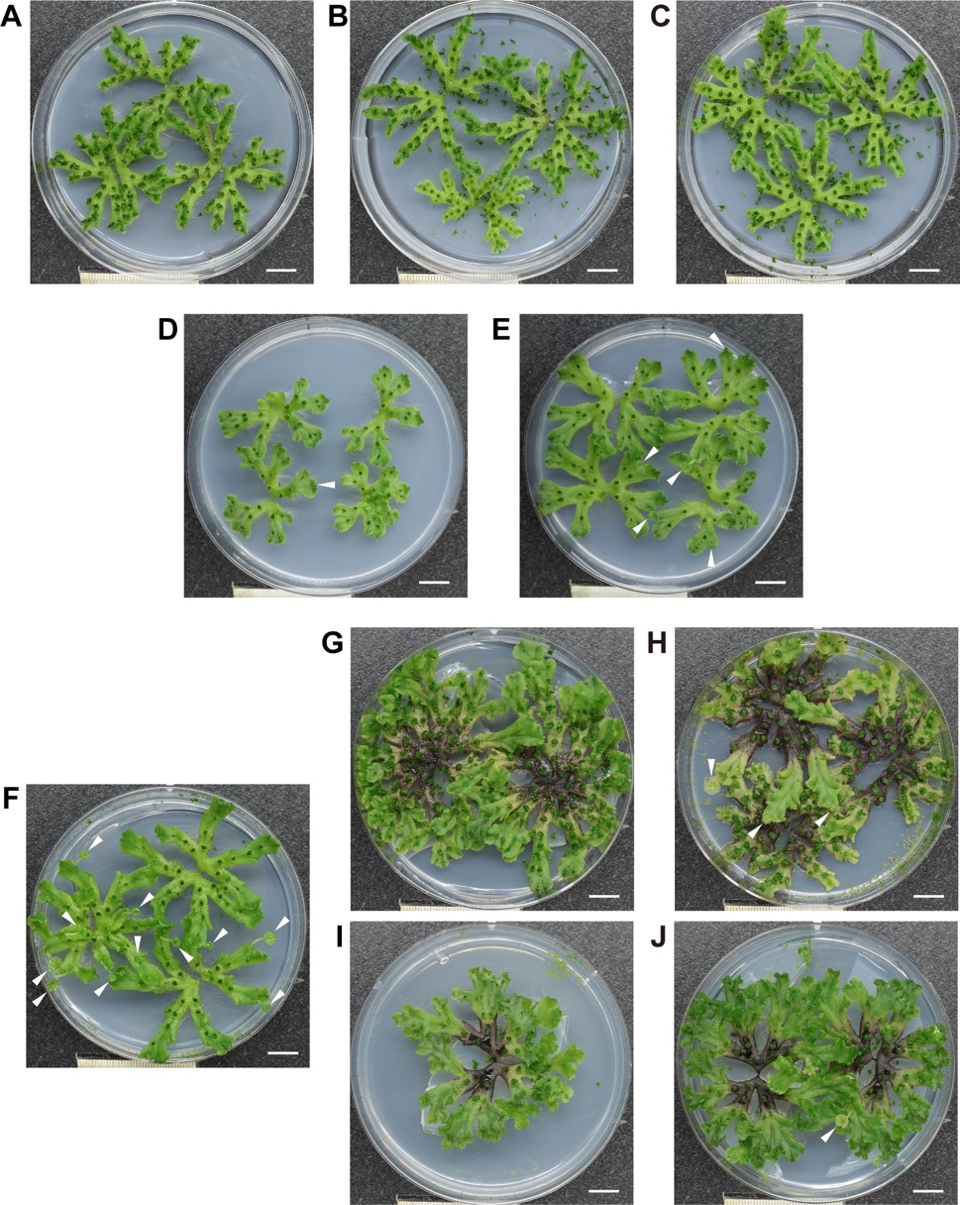
­­­­­

­

­­

**Supplementary Figure 5. Representative plants grown in non 24-h light/dark cycles.**

(A) to (C) Images of representative plants grown for about 60 astronomical days (i. e. in 24-h day) under conditions of T-cycles comprised of light and dark periods in a ratio of 1 to 2 (Fig. 4A). 6L12D (A), 10L20D (B), and 12L24D (C). An image of a representative plant grown in 8L16D conditions is shown in Fig. 1B. (D) and (E) Images of representative plants grown for 28 astronomical days under 8L10D (D) and 8L8D (E) conditions in a separate set of experiments similar to those shown in Fig. 4B. In this set of experiments terminated on 28th day, the number of astronomical days required for the formation of the first visible gametangiophore primordium was 25 to 28 astronomical days in 8L8D (n = 8) and the first gametangiophore primordium was observed in one plant (among 8 plants) on 28th astronomical day in 8L10D. (F) to (J) Images of representative plants grown under 16L16D, 24L24D, and 36L24D conditions (**Fig. 4C**). A plant grown for 33 days in 16L16D conditions with gametangiophores (F) and plants grown for about 60 days in 24L24D (G, H) and 36L24D (I, J). For 24L24D and 36L24D conditions, representative plants with no gametangiophore development (G, I) or with developing gametangiophores (H, J) are shown. Arrowheads indicate developing gametangiophores. Dark green spots on the thallus are gemma cups filled with gemmae. Scale bars = 1 cm.
